# New systemic treatment paradigms in advanced biliary tract cancer and variations in patient access across Europe

**DOI:** 10.1016/j.lanepe.2024.101170

**Published:** 2025-02-19

**Authors:** Lorenza Rimassa, Angela Lamarca, Grainne M. O'Kane, Julien Edeline, Mairéad G. McNamara, Arndt Vogel, Matteo Fassan, Alejandro Forner, Timothy Kendall, Jorge Adeva, Andrea Casadei-Gardini, Lorenzo Fornaro, Antoine Hollebecque, Maeve A. Lowery, Teresa Macarulla, David Malka, Elene Mariamidze, Monica Niger, Anu Ustav, John Bridgewater, Rocio I.R. Macias, Chiara Braconi

**Affiliations:** a Department of Biomedical Sciences, Humanitas University, Via Rita Levi Montalcini 4, Pieve Emanuele, Milan, 20072, Italy; b Medical Oncology and Hematology Unit, Humanitas Cancer Center, IRCCS Humanitas Research Hospital, Via A. Manzoni 56, Rozzano, Milan, 20089, Italy; c Department of Medical Oncology, Oncohealth Institute, Instituto de Investigación Sanitaria de la Fundación Jiménez Díaz, Fundación Jimenez Diaz University Hospital, Avda Reyes Católicos 2, Madrid, 28040, Spain; d University College Dublin, Belfield, Dublin 4, Ireland; e Department of Medical Oncology, St. Vincent's University Hospital, Elm Park, Dublin 4, Ireland; f INSERM, Department of Medical Oncology, University Rennes, CLCC Eugène Marquis, COSS [(Chemistry Oncogenesis Stress Signaling)] – UMR_S 1242, Rennes, F-35000, France; g Division of Cancer Sciences, University of Manchester & Department of Medical Oncology, The Christie NHS Foundation Trust, Manchester, M20 4BX, UK; h Toronto General Hospital, UHN, 200 Elizabeth Street, Toronto, ON, M5G 2C4, Canada; i Princess Margaret Cancer Centre, 610 University Ave, Toronto, ON, M5G 2M9, Canada; j Hannover Medical School, Carl-Neuberg Str. 1, Hannover, 30659, Germany; k Department of Medicine (DIMED), University of Padua, Via Gabelli 61, Padua, 35121, Italy; l Veneto Institute of Oncology (IOV-IRCCS), Via Gattamelata 64, Padua, 35128, Italy; m Liver Unit, Barcelona Clinic Liver Cancer (BCLC) Group, ICMDM, Hospital Clinic IDIBAPS, University of Barcelona, Villarroel 170, Barcelona, 08036, Spain; n Centro de Investigación Biomédica en Red de Enfermedades Hepáticas y Digestivas (CIBERehd), Av. Monforte de Lemos, 3-5, Madrid, 28029, Spain; o Centre for Inflammation Research, Institute for Regeneration and Repair, University of Edinburgh, 5 Little France Drive, Edinburgh, EH16 4UU, UK; p Edinburgh Pathology, University of Edinburgh, 51 Little France Crescent, Edinburgh, EH16 4SA, UK; q CRUK Scotland Cancer Centre, Switchback Rd, Glasgow, G61 1BD, UK; r Department of Medical Oncology, Hospital Universitario 12 de Octubre, Av. de Córdoba, s/n, Usera, Madrid, 28041, Spain; s Department of Oncology, Vita-Salute San Raffaele University, IRCCS San Raffaele Scientific Institute Hospital, Via Olgettina 60, Milan, 20132, Italy; t Medical Oncology 2 Unit, Azienda Ospedaliero-Universitaria Pisana, Via Roma 67, Pisa, 56126, Italy; u Département de Médecine Oncologique, Gustave Roussy, 114 Rue Edouard Vaillant, Villejuif, F-94805, France; v Trinity St James Cancer Institute, Trinity College Dublin, College Green, Dublin 2, Ireland; w Vall d'Hebrón Institute of Oncology (VHIO), Vall d'Hebrón University Hospital, Centre Cellex, Carrer de Natzaret, 115-117, Barcelona, 08035, Spain; x Department of Medical Oncology, Institut Mutualiste Montsouris, 42 Boulevard Jourdan, Paris, 75014, France; y Department of Oncology and Hematology, Todua Clinic, Tevdore Mgvdeli #13, Tbilisi, 0112, Georgia; z Medical Oncology Department, Fondazione IRCCS Istituto Nazionale dei Tumori di Milano, Via Venezian 1, Milan, 20133, Italy; aa Clinic of Oncology, North-Estonian Medical Centre, Sytiste Rd 19, Tallinn, 13419, Estonia; ab UCL Cancer Institute, 72 Huntley St, London, WC1E 6DD, UK; ac Experimental Hepatology and Drug Targeting (HEVEPHARM) Group, University of Salamanca, IBSAL, CIBERehd, Campus M. Unamuno s/n, Salamanca, 37007, Spain; ad School of Cancer Sciences, University of Glasgow, Switchback Rd, Glasgow, G61 1QH, UK; ae Beatson West of Scotland Cancer Centre, 1053 Great Western Rd, Glasgow, G12 0YN, UK

**Keywords:** Biliary tract cancer, Cholangiocarcinoma, Gallbladder cancer, Immunotherapy, Molecularly targeted therapy, NGS, Access to therapy, Access to NGS

## Abstract

In recent years, treatment options for patients with advanced biliary tract cancer (BTC) have increased significantly due to the positive results from phase 2/3 clinical trials of immune checkpoint inhibitors, combined with chemotherapy, and molecularly targeted agents. These advances have led to the need for molecular testing to identify actionable alterations and patients amenable to targeted therapies. However, these improvements have brought with them many questions and challenges, including the identification of resistance mechanisms and therapeutic sequences. In this Series paper we aim to provide an overview of the current systemic treatment options for patients with BTC, highlighting disparities in access to innovative treatments and molecular testing across European countries, which lead to inequalities in the possibilities of treating patients with advanced BTC. We also discuss how ongoing European collaborative projects, such as the COST Action Precision-BTC-Network CA22125, supported by COST (European Cooperation in Science and Technology), linked to the European Network for the Study of Cholangiocarcinoma (ENSCCA), can help overcome these disparities and improve the current scenario.

## Introduction

Chemotherapy has long been the mainstay of systemic treatment for patients with advanced biliary tract cancer (BTC), based on phase 3 data.^1^^,^^2^ The ABC-02 trial demonstrated that the combination of cisplatin and gemcitabine (CisGem) improved outcomes compared to single-agent gemcitabine,^1^ and the ABC-06 trial showed the benefit of the combination of folinic acid, 5-fluorouracil (5-FU), and oxaliplatin (mFOLFOX) versus active symptom control after CisGem.^2^

**Key messages**

• Treatment for advanced BTC has significantly improved in recent years with over ten new therapies, but their reimbursement varies across Europe
• Molecular profiling is crucial to identify actionable alterations and define the correct treatment pathway; identifying resistance mechanisms and therapeutic sequences is still challenging significant disparities in healthcare systems and reimbursement lead to inequalities in access to innovative treatments and molecular testing across European countries. International collaboration is needed to harmonise treatment access and approval processes
• It is essential that different stakeholders and government bodies become aware of these challenges and advocate to drive policy change and overcome these disparities

**Search strategy and selection criteria**

References for this Series paper were identified through searches of PubMed with the search terms “biliary cancers”, “treatment”, “genomic profiling” from 2010 until 2024. Articles were also identified through searches of the authors’ own files. Only papers published in English were reviewed. The final reference list was generated on the basis of originality and relevance to the broad scope of this Series paper.

Recently, new therapeutic options, such as immune checkpoint inhibitors (ICIs) and molecularly targeted agents, have improved outcomes while preserving quality of life in patients with advanced BTC. Two phase 3 trials demonstrated that the combination of CisGem with ICIs, durvalumab or pembrolizumab, improved survival compared to CisGem.^3^, ^4^, ^5^, ^6^, ^7^, ^8^ Furthermore, the discovery of genomic alterations paved the way for precision medicine, and the emergence of molecularly targeted agents is rapidly changing the therapeutic approach for patients with BTC. Phase 2 and 3 studies have shown the benefit of pemigatinib and futibatinib for patients with *FGFR2* gene fusions/rearrangements,^9^, ^10^, ^11^ and of ivosidenib for patients with *IDH1* mutations previously treated with chemotherapy,^12^^,^^13^ while other phase 1/2 studies have identified further therapeutic options for patients with other molecular alterations.^14^, ^15^, ^16^

However, these positive results have brought many questions and challenges, including the identification of resistance mechanisms and therapeutic sequences. The European Medicines Agency (EMA) has approved durvalumab and pembrolizumab in combination with CisGem for first line treatment of patients with BTC; pemigatinib, futibatinib, and ivosidenib for previously treated patients with appropriate molecular alterations; and additional agnostic approvals for targeted therapies are relevant for patients with BTC. However, access to these drugs is not homogenous within Europe and differences in the healthcare systems across European countries lead to significant disparities in the available treatments for these patients.

**Fig. 1:**
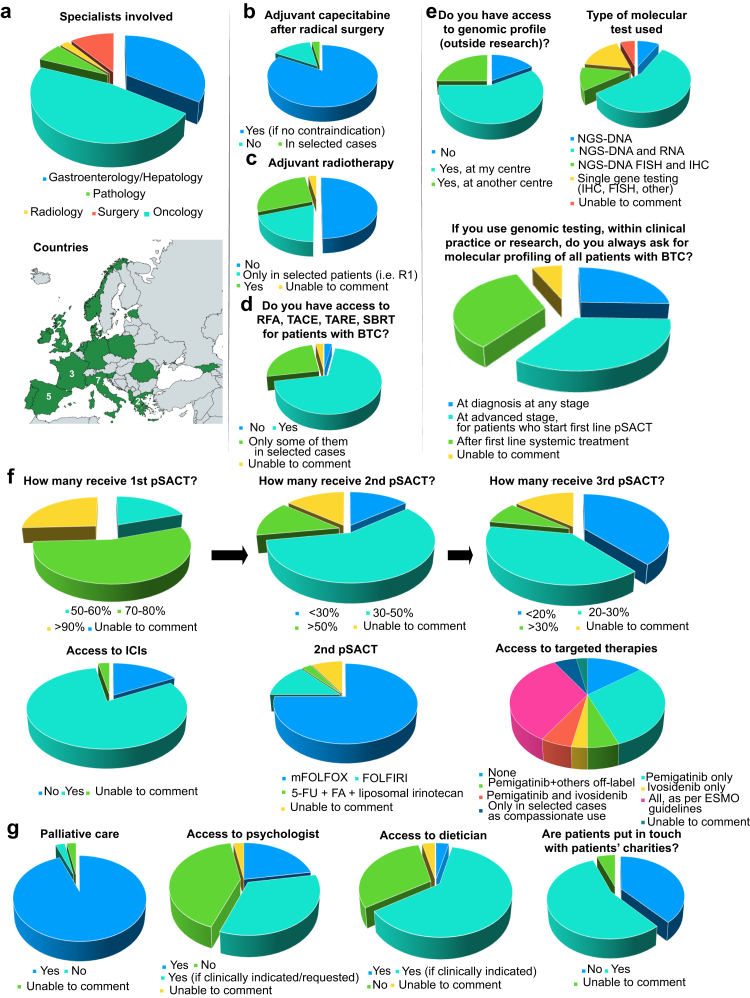
**Results of the survey on clinical practice in biliary tract cancer in Europe.** Panel a provides information regarding the survey's participants (n = 47 physicians). If more than one physician from a specific country answered to the survey, the exact number of participants from that country is reported on the map. Answers regarding access to systemic and locoregional therapies are reported in panels b, c, d, and f; answers regarding access to genomic profiling are reported in panel e. Panel g provides information regarding access to supportive care. All answers provided in panels b, c, d, e, f, and g are divided by institution (n = 36). Abbreviations: 5-FU, 5-fluorouracil; BTC, biliary tract cancer; ESMO, European Society of Medical Oncology; FA, folinic acid; FISH, fluorescence *in situ* hybridisation; FOLFIRI, folinic acid + 5-FU + irinotecan; FOLFOX, folinic acid + 5-FU + oxaliplatin; ICIs, immune checkpoint inhibitors; IHC, immunohistochemistry; NGS, next-generation sequencing; pSACT, palliative systemic anticancer treatment; RFA, radiofrequency ablation; SBRT, stereotactic body radiation therapy; TACE, transarterial chemoembolisation; TARE, transarterial radioembolisation. Created with Adobe Illustrator.

In this series paper we provide an overview of current systemic treatments for patients with BTC, and highlight the recent improvements and revision of guidelines. We have also conducted a survey across 47 European physicians on the implementation of novel technologies and drugs. The survey included 24 questions and was sent to the co-authors of this manuscript and other key professionals involved in the management of patients with BTC. Participants included oncologists (n = 22), hepatologists/gastroenterologists (n = 16), surgeons (n = 5), pathologists (n = 3), and radiologists (n = 1). 36 institutions were represented across 18 countries (Fig. 1 and Supplementary Material, Table S1). We have identified challenges in access to innovative therapies in European countries, underline the need to address inequalities, and present ongoing European projects aiming to improve the current scenario.

## Chemotherapy for the treatment of advanced BTC

Until recently, CisGem was the standard first line option in advanced unresectable or metastatic BTC. The ABC-02 trial demonstrated a median overall survival (OS) of 11.7 months with CisGem versus 8.1 months with gemcitabine alone.^1^ Attempts to improve survival outcomes with triplet chemotherapy regimens have been unsuccessful in Western populations.^17^, ^18^, ^19^, ^20^ Both the PRODIGE 38 AMEBICA trial of mFOLFIRINOX and the SWOG1815 trial of CisGem and albumin-bound paclitaxel failed to show improvements in OS over CisGem,^17^, ^18^, ^19^ although in the later study, patients with locally advanced disease and gallbladder cancer (GBC) appeared to benefit from the triplet. The Japanese KHBO1401-MITSUBA randomised phase 3 trial documented higher objective response rate (ORR) (41% versus 15%) and improved median OS (13.5 versus 12.6 months) when comparing CisGem/S1 versus CisGem alone; notably, two-thirds of patients had GBC or extrahepatic cholangiocarcinoma (eCCA).^21^ In countries where the addition of ICIs is yet to be approved and reimbursed, CisGem remains the standard first line regimen.

Following progression on first line therapy, in the absence of targetable alterations, second line chemotherapy has been shown to improve survival and can be considered in patients who remain fit.^22^ The ABC-06 trial was a phase 3 study randomising patients who had progressed on CisGem to mFOLFOX versus active symptom control.^2^ This study showed an OS benefit with chemotherapy (median OS of 6.2 months versus 5.3 months). Median progression-free survival (PFS) was 4 months, with partial response rate of 5%. Despite modest benefit, quality of life was not worsened in the chemotherapy arm, therefore it is considered a safe treatment in this setting.^23^ There are, however, no biomarkers for selection of patients for mFOLFOX, and all patients appear to benefit similarly, regardless of response to prior platinum or status of genes associated with DNA damage repair.^24^

The combination of 5-FU and liposomal irinotecan has been explored as a potential alternative to mFOLFOX. However, conflicting results have been reported. First, the NIFTY South Korean phase 2 randomised trial reported an improved OS over 5-FU alone (8.6 months versus 5.3 months); PFS was similar (4.2 months) to the one reported for mFOLFOX in the ABC-06 trial.^25^, ^26^ In contrast, the German NALIRICC trial, with a similar study design, did not reach significance: median OS was 8.2 months versus 6.9 months, and median PFS was 2.6 months versus 2.3 months.^27^ In both studies, ORR was approximately 12–14%.

Based on these data, guidelines recommend mFOLFOX in the absence of targetable alterations. In selected patients with contraindications to mFOLFOX, 5-FU and irinotecan could be considered as an alternative choice, though there are no robust data to support this.^14^

According to our survey, chemotherapy is widely accessible across different countries in Europe. In the first line setting, CisGem is widely accessible, with more than 80% of institutions also having access to ICIs. However, it is interesting to note that many patients are not fit to start a first line palliative systemic anticancer treatment (pSACT), with most institutions prescribing first line pSACT in less than 70% of patients with a new diagnosis of unresectable BTC, suggesting that there is scope for improving supportive care at diagnosis. In the second line setting, current practice highlights that in 70% of institutions less than 50% of patients with BTC receive second line pSACT due to rapid deterioration of their performance status. Importantly, the figures in our survey are in line with previously published data. In two analyses of the French nation-wide hospitalisation database, accounting for 3650 patients with intrahepatic CCA (iCCA)^28^ and 19,825 patients with BTC respectively, only 812 (22%) patients and 7721 (38.9%) patients received pSACT.^29^ mFOLFOX represents the predominant standard of care in Europe, and in 75% of institutions physicians prescribe it over FOLFIRI or liposomal irinotecan/5-FU (Fig. 1).

## EMA approved chemoimmunotherapy combinations in first line

The use of ICIs for the treatment of advanced BTC has been explored. Except for the small subgroup of patients with microsatellite instability-high (MSI-H) tumours, the results of anti-programmed death-1 (PD-1) single agents were disappointing (ORR 3–13%).^30^, ^31^, ^32^ However, results of combination with chemotherapy as first line treatment were more promising. In a phase 2 trial in South Korea testing different regimens of chemotherapy plus durvalumab [an anti-programmed death-ligand 1 (PD-L1) antibody] with or without tremelimumab (an anti-cytotoxic T lymphocyte antigen-4 antibody) ORR ranged from 50% to 72% and median OS from 24.2 to 26.6 months.^33^

The TOPAZ-1 was a phase 3 trial, which compared the combination of CisGem with durvalumab versus CisGem with placebo in the first line setting. The primary endpoint of OS was met, with a median OS of 12.9 versus 11.3 months.^3^^,^^4^ Secondary endpoints of PFS (7.2 months versus 5.2 months) and ORR (27% versus 19%) were also improved; 2% of patients experienced grade 3/4 immune-mediated adverse events (imAEs) in the durvalumab arm versus 1% in the placebo arm. Recently, multiple real-world studies of the CisGem-durvalumab combination confirmed the results of this trial and the safety of this combination.^34^, ^35^, ^36^ The KEYNOTE-966 phase 3 trial demonstrated the benefit of pembrolizumab (an anti-PD-1 antibody) combined with CisGem over CisGem alone in this setting.^6^^,^^7^ The main difference in design with TOPAZ-1 was the possibility of gemcitabine maintenance after the 6 months of CisGem. KEYNOTE-966 showed a significant improvement in the primary endpoint of OS (median of 12.7 versus 10.9 months). The efficacy threshold for PFS benefit was not met according to the statistical design of the trial, possibly due to the maintenance gemcitabine that might have improved results in the control arm. Grade 3/4 imAEs were seen in 7% of the patients in the pembrolizumab arm versus 4% in the placebo arm.

Of note, neither TOPAZ-1 nor KEYNOTE-966 could define a subgroup of patients who would benefit more from the addition of ICIs, even based on PD-L1 expression. Overall, these phase 3 studies show a modest benefit in OS, but unprecedented 1-, 2-, and 3-year OS rates with chemoimmunotherapy as first line treatment, without undue increase of toxicity. Thus, these combinations are now standard of care (Table 1) and both durvalumab and pembrolizumab are approved by the EMA in combination with CisGem as first line treatment of advanced BTC. Nonetheless, in our survey, physicians from six different institutions (n = 6 countries, 16%) commented that neither were available in their centre (Fig. 1).

**Table 1:** Key phase 3 trials of first line treatments for advanced biliary tract cancers.

	ABC-02^1^		TOPAZ-1^3^, ^4^, ^5^		KEYNOTE-966^6^, ^7^, ^8^	
	CisGem (n = 204)	Gem (n = 206)	CisGem + durva (n = 341)	CisGem + placebo (n = 344)	CisGem + pembro (n = 533)	CisGem + placebo (n = 536)
Treatment duration	8 cycles	6 cycles	8 cycles followed by maintenance with durva	8 cycles followed by maintenance with placebo	8 cycles followed by maintenance with gem + pembro for 2 years	8 cycles followed by maintenance with gem + placebo for 2 years
Median OS, months (95% CI)	11.7 (9.5–14.3)	8.1 (7.1–8.7)	12.9 (11.6–14.1)	11.3 (10.1–12.5)	12.7 (11.5–13.6)	10.9 (9.9–11.6)
OS: HR (95% CI) p	0.64 (0.52–0.80) <0.001		0.76 (0.64–0.91) NA		0.86 (0.75–0.98) 0.0099	
1-year OS rate, % (95% CI)	NA	NA	54.3 (48.8–59.4)	47.2 (41.7–52.4)	52 (NA)	44 (NA)
2-year OS rate, % (95% CI)	NA	NA	23.6 (18.7–28.9)	13.1 (9.8–17.0)	24.6 (21.0–28.3)	19.2 (16.0–22.6)
3-year OS rate, % (95% CI)	NA	NA	14.6 (11.0–18.6)	6.9 (4.5–10.0)	13 (NA)	11 (NA)
Median PFS, months (95% CI)	8.0 (6.6–8.6)	5.0 (4.0–5.9)	7.2 (6.7–7.4)^a^	5.7 (5.6–6.7)^a^	6.5 (5.7–6.9)	5.6 (4.9–6.5)
PFS: HR (95% CI) p	0.63 (0.51–0.77) <0.001		0.75 (0.63–0.89)^a^ 0.001^a^		0.85 (0.75–0.97) NA	
ORR, %	NA	NA	27	19	28.7	28.7
DCR, %	81.4	71.8	85.3^a^	82.6^a^	NA	NA
Median DOR, months (range)	NA	NA	6.4 (4.6–17.2)^a^	6.2 (3.8–9.0)^a^	8.3 (1.2+ –44.3+)	6.9 (1.1+ –41.1+)
Safety	n = 198	n = 199	n = 338	n = 342	n = 529	n = 534
G3–4 AEs, %	70.7	68.8	74	75	79.4	74.7
Led to discontinuation of ≥1 study medication, %	NA	NA	13	15	26.5	23.2
Led to death, %	NA	NA	4	4	5.9	9.4
Quality of life						
QLQ-C30	NA	NA	n = 318	n = 328	n = 518	n = 517
QLQ-BIL21	NA	NA	n = 305	n = 322	n = 518	n = 516
Median time to deterioration of global health status or quality of life, months (95% CI)	NA	NA	7.4 (5.6–8.9)	6.7 (5.6–7.9)	Not reached (NA)	21.2 (NA)
HR (95% CI)	NA	NA	0.87 (0.69–1.12)		0.86 (0.70–1.07)	
Adjusted mean change from baseline (95% CI)	NA	NA	1.23 (0.71–3.16)	0.35 (−1.63 to 2.32)	−2.5 (−4.5 to −0.5)	−2.5 (−4.5 to −0.5)

Abbreviations: AEs, adverse events; CI, confidence interval; CisGem, cisplatin and gemcitabine; DCR, disease control rate; DOR, duration of response; durva, durvalumab; G, grade; gem, gemcitabine; HR, hazard ratio; n, number; NA, not available; pembro, pembrolizumab; ORR, objective response rate; OS, overall survival; PFS, progression-free survival.

a Data from the interim analysis.^3^

## EMA approved molecularly targeted agents in second line and beyond

Options for treatment of patients with advanced BTC beyond first line include molecularly targeted therapies, and in up to 50% of patients with iCCA druggable alterations may be found (Table 2).^14^^,^^37^ However, availability in clinical practice is influenced by many factors, including access to molecular profiling, drug approval and reimbursement.

**Table 2:** Targeted therapies for advanced biliary tract cancers.

Gene	Type of alteration	Frequency (%)	Drug	Phase of trial	ORR (%)	DCR (%)	Median PFS (months)	Median OS (months)
*FGFR2*	Rearrangement or fusion	10–16	Pemigatinib^9^^,^^10^ Futibatinib^11^ Infigratinib^38^^,^^39^ Derazantinib^40^ Lirafugratinib (RLY-4008)^41^ Tinengotinib^42^^,^^43^	2 2 2 2 1/2 2	37.0 42.0 23.1 21.4 52/14 9.1/37.5	82.4 83.0 84.3 75.7 88/80 94.7	7.0 9.0 7.3 8.0 NA 26	17.5 21.7 12.2 17.2 NA NA
*IDH1*	Mutation	10–15	Ivosidenib^12^^,^^13^	3	2.0	53.0	2.7	10.3
*HER2*	Amplification or overexpression	eCCA 20 iCCA 5	Trastuzumab + mFOLFOX^44^ Trastuzumab + pertuzumab^45^ Zanidatamab^46^ Trastuzumab deruxtecan^47^ Tucatinib + trastuzumab^48^	2 2 2b 2 2	29.4 23.0 41.3 22.0 46.7	NA 51.0 68.8 65.9 76.7	5.1 4.0 5.5 4.6 5.5	10.7 10.9 5.5 7.0 NA
*HER2*	Mutation	2	Neratinib^49^	2	16	28	2.8	5.4
*BRAF*	Class I mutation	3	Dabrafenib + trametinib^50^ Vemurafenib + cobimetinib^51^^,^^a^	2 2	53.0 57	NA 68	9.0 23 weeks	13.5 61 weeks
dMMR	Inactivating alteration of MLH1, MSH2, MSH6, or PMS2	1–2	Pembrolizumab^31^^,^^32^	2	40.9	NA	4.2	19.4
*NTRK*	Rearrangement	<1	Entrectinib^52^ Larotrectinib^53^	1/2 1/2	57.0 79.0	NA NA	11.2 28.3	21.0 44.4
*RET*	Rearrangement	<1	Selpercatinib^54^ Pralsetinib^55^	1/2 1/2	43.9 66	NA NA	13.2 NA	NA NA
*KRAS*	G12C mutation	<1	Adagrasib^56^	2	41.7	91.7	8.6	15.1

Abbreviations: DCR, disease control rate; eCCA, extrahepatic cholangiocarcinoma; iCCA, intrahepatic cholangiocarcinoma; NA, not available; ORR, objective response rate; OS, overall survival; PFS, progression-free survival.

a Only two patients with biliary tract cancer were included; data are for the entire study population.

*FGFR2* fusions/rearrangements are found in 10–16% of patients with iCCA.^37^^,^^57^ In 2020, pemigatinib showed efficacy in patients with previously treated CCA with *FGFR2* fusions/rearrangements, with an ORR of 37%, a DCR of 82.4%, a median PFS of 7 months, and a median OS of 17.5 months.^9^^,^^10^ These results were confirmed in a real-life setting as well.^58^^,^^59^ Pemigatinib was designated an “orphan medicine” in 2018 (EU/3/18/2066) and was granted EMA approval (conditional authorisation) in 2021. More recently, futibatinib has demonstrated clinical activity in pretreated patients with *FGFR2* fusions/rearrangements, yielding an ORR of 42% and a DCR of 83%; median PFS was 9 months, and median OS was 21.7 months.^11^ Based on these results, it was conditionally authorised by the EMA in 2023. Additional FGFR-targeted agents have reported clinical benefit in patients with pretreated CCA harbouring *FGFR2* fusions/rearrangements: infigratinib,^38^^,^^39^ derazantinib,^40^, ^60^ lirafugratinib,^41^ and tinengotinib.^42^^,^^43^ Both infigratinib and derazantinib are not approved by European regulatory entities, with infigratinib's marketing authorisation being withdrawn in 2022. Lirafugratinib and tinengotinib were designated orphan medicines for BTC by the EMA in 2022 and 2024, respectively. However, in 2023 it was announced that the lirafugratinib programme for the treatment of patients with BTC was paused due to the financial impact of the Inflation Reduction Act.^61^ It is noteworthy that the increasing number of agents in the same regulatory space may have repercussions on the marketing strategy of different pharmaceutical companies. Considering the efficacy of FGFR inhibitors, attempts have been made to assess their efficacy in first line, but both the FOENIX-CCA3 trial of futibatinib versus CisGem (NCT04093362), and the PROOF 301 trial of infigratinib versus CisGem (NCT03773302) have been discontinued due to slow accrual.^62^ The FIGHT-302 randomised phase 3 trial aims to further assess the effectiveness and safety of pemigatinib in the first line setting, but even in this case patients accrual may represent a limitation.^63^

*IDH1* mutations are found in 10–15% of patients with iCCA.^37^^,^^64^^,^^65^ In 2020, in the ClarIDHy phase 3 trial—the only completed phase 3 trial to date assessing a molecularly targeted agent in BTC—ivosidenib demonstrated clinical benefit in patients with *IDH1*-mutated CCA who had received up to two previous treatments for advanced disease (median OS 10.3 months, median PFS 2.7 months).^12^^,^^13^ Analogous outcomes were observed in real-world series.^66^^,^^67^ Ivosidenib obtained orphan designation in 2018 (EU/3/18/1994) and was EMA approved in 2023 for patients with pretreated CCA and *IDH1 R132* mutations.

*HER2* is amplified or overexpressed in 20% of patients with eCCA and 5% of patients with iCCA, while mutations are more rare.^37^^,^^68^ A phase 2 single-arm trial of second line trastuzumab/FOLFOX yielded a median PFS of 5.1 months, an ORR of 29.4%, and an OS of 10.7 months in patients with HER2-positive BTC.^44^ Other promising agents targeting HER2 for patients with pretreated BTC include pertuzumab/trastuzumab (ORR 23%),^45^ zanidatamab (ORR 41.3%),^46^ trastuzumab deruxtecan (ORR 37.1%),^47^ and tucatinib/trastuzumab (ORR 46.7%).^48^ Zanidatamab received EMA orphan drug designation (EU/3/21/2458) and priority review by the United States (US) Food and Drug Administration (FDA) for the treatment of BTC. In 2024, the FDA granted tumour agnostic accelerated approval to trastuzumab deruxtecan for the treatment of patients with pretreated advanced HER2-positive (immunohistochemistry [IHC] 3+) solid tumours without alternative therapeutic options. By contrast, it received EMA conditional marketing authorisation (with additional monitoring) only for the treatment of patients with selected HER2-positive tumour types, not including BTC.

*BRAF* mutations are found in 9% of patients with BTC, with *BRAF* Class I mutations occurring in roughly 3%.^37^^,^^50^^,^^69^ In a phase 2, single-arm, Rare Oncology Agnostic Research basket trial, dabrafenib (BRAF inhibitor) plus trametinib (MEK inhibitor) showed promising activity (independent reviewer-assessed ORR 47%) and a manageable safety profile in patients with *BRAF V600E*-mutated pretreated BTC.^50^ Dabrafenib/trametinib has received tumour agnostic approval from the FDA. EMA marketing authorisation applies to *BRAF V600*-mutated advanced melanoma, non-small cell lung cancer (NSCLC), low-grade glioma, and BTC in selected countries. For instance, from July 2024 dabrafenib/trametinib is available in Italy for patients with previously treated *BRAF V600E*-mutated advanced BTC within a national public compassionate use (“Legge 648”). More recent data support the activity of cobimetinib plus vemurafenib in an agnostic fashion showing promising ORR (57%) in solid tumours (n = 31) with *BRAF V600E* mutations; however EMA marketing authorisation is currently available only for melanoma.^51^

Pembrolizumab initially showed clinical benefit in patients with mismatch repair-deficient (dMMR) non-colorectal cancer, including four patients with CCA or ampullary cancer (ORR 71%).^70^ This data was confirmed in the KEYNOTE-158 trial (22 patients with pretreated CCA [ORR 40.9%]).^32^^,^^33^ Thus, from 2022 it received EMA marketing authorisation for patients with pretreated MSI-H or dMMR advanced tumours, including BTC. Typically, tumour mutational burden (≥10 mutations/megabase) is also used to guide access to ICIs, but it has failed to demonstrate predictive value in patients with BTC.^3^^,^^4^^,^^6^^,^^7^

*NTRK* and *RET* gene fusions are rare in BTC (less than 1% each).^37^^,^^71^, ^72^, ^73^ Larotrectinib and entrectinib (anti-NTRK drugs) have EMA conditional marketing authorisations in patients with solid tumours harbouring an *NTRK* gene fusion, while selpercatinib (anti-RET agent) has EMA conditional marketing authorisation for pretreated patients with *RET* fusions and no other therapeutic options.

*KRAS*^G12C^ mutations are present in 1.1% of BTC and can be targeted by selected KRAS inhibitors.^37^ Sotorasib has marketing authorisation for NSCLC; lastly, adagrasib has conditional marketing EMA authorisation only for patients with advanced *KRAS*^*G12C*^ mutated NSCLC, despite its efficacy data.^56^ In 2010–2019, of 89 new oncology therapies, the FDA approved 95% before European authorisation.^74^ Additionally, the median (IQR) time for review for the FDA was 200 days (155–277) versus 426 days (358–480) for the EMA,^74^ beyond the expected prognosis of approximately one year for patients with advanced BTC.^3^, ^4^, ^5^, ^6^, ^7^, ^8^ Early access programmes may be a solution to enable earlier access to investigational drugs in Europe.^75^ Drug manufacturers submit regulatory documents earlier to the FDA than to the EMA, probably due to the greater market share and higher cost of drugs in the US.^76^

While access to medicines for rare diseases such as BTC has to be a priority, the requisite for well-designed clinical trials (e.g., basket trials, use of synthetic controls) to provide evidence of safety and efficacy at affordable sustainable prices is paramount. So far, all the targeted drugs currently available for BTC have been tested either in single arm studies or in basket trials, with the exception of ivosidenib, where the control arm was placebo. In 2021, the ABC-06 trial defined mFOLFOX as the standard of care in second line and set the benchmark for OS at 6.2 months. Given all of these trials pre-dated the ABC-06 study, none of the regulatory studies for targeted therapies included mFOLFOX as the appropriate comparator. Even though the lack of head-to-head comparison makes the interpretation of the results more challenging, the remarkable activity in clinical benefit, reflected also in extended median OS (ranging from 10.3 to 21.7 months with targeted therapies), has been at the base of the approvals.

**Fig. 2:**
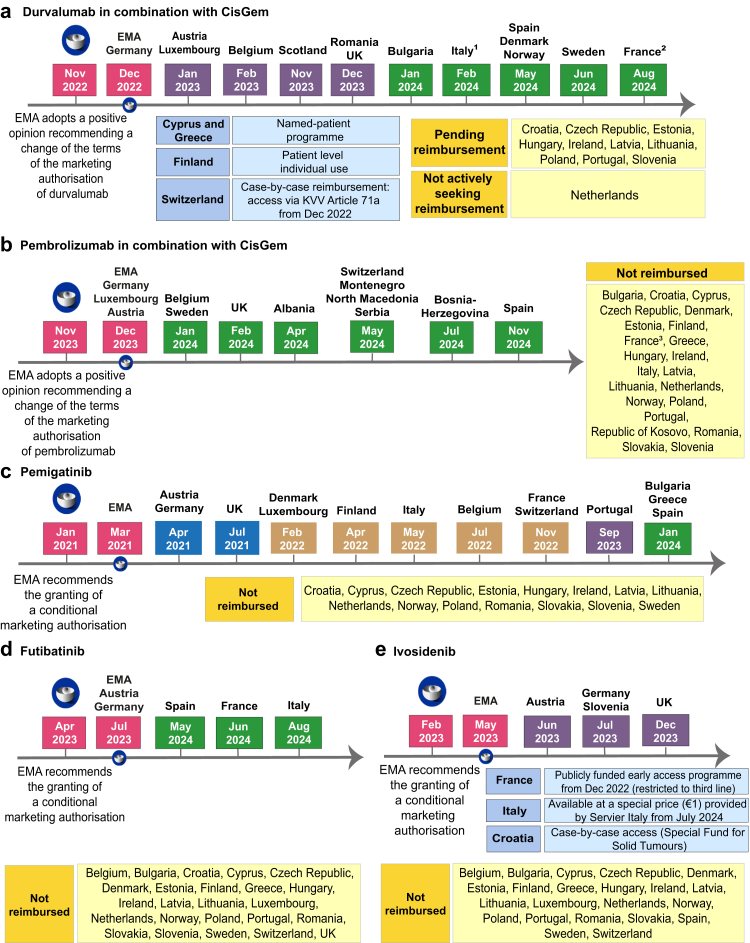
**Timeline of approvals of durvalumab (a) and pembrolizumab (b) in combination with chemotherapy, pemigatinib (c), futibatinib (d), and ivosidenib (e) for the treatment of biliary tract cancer in Europe.**^1^From March 21st, 2022 until February 18th, 2024, durvalumab in association with chemotherapy was available in Italy via a named-patient programme provided by AstraZeneca Italy. ^2^From October 2022 until July 31st, 2024 durvalumab in association with chemotherapy was available in France via a publicly funded early access programme in the ITT population. ^3^In France publicly funded early access to pembrolizumab in association with chemotherapy was denied on March 21st, 2023.^4^ In 2020 and 2021 pemigatinib was available in France via publicly funded early access (ATU). Abbreviations: ATU, temporary use authorisation; CisGem, cisplatin and gemcitabine; EMA, European Medicines Agency; ITT, intention-to-treat; KVV, Verordnung über die Krankenversicherung (Ordinance on Health Insurance); UK, United Kingdom. Created with Adobe illustrator.

The additional challenge in time to reimbursement for these drugs should also be acknowledged. The time limit for reimbursement of new anti-cancer medicines in 2016–2021 (180 days), as recommended by the Council of European communities following European Union Market Access directive, was met for 100% of included medicines by Germany, 51% by France, 29% by the United Kingdom (UK) and the Netherlands, 14% by Switzerland, 6% by Norway, and 3% by Belgium.^77^ It was reported that factors associated with shorter time to reimbursement included higher gross domestic product (GDP), absence of pre-assessment procedures, and submission by a big pharmaceutical company.^77^ In this study, there was inequality in access to these drugs between these seven high-income countries, which is likely only a fraction of the inequality between these countries and European countries of lower GDP. Furthermore, according to our survey, patients have access to all EMA-approved targeted agents in only one third of the institutions (Fig. 1). The delay in the approval of durvalumab, pembrolizumab, pemigatinib, futibatinib, and ivosidenib in many European countries, despite EMA authorisation, is a key example of the differences in access to anticancer therapies across Europe (Fig. 2).

While efficacy and safety have to be maintained, expedient, efficient, and affordable European access is essential. To drive policy change, it is mandatory that treating clinicians, the general oncology community, informed patient advocacy groups and governing bodies become more aware and vocal about these challenges and echo them.

## European guidelines for molecular profiling

Molecular profiling is essential to inform treatment options for patients with advanced BTC being considered for systemic therapy and should be performed at the earliest opportunity. Multiple experiences in tumour agnostic trials have provided evidence for the clinical benefit of a precision oncology approach based on genomic profiling compared to an all-comer strategy, as demonstrated by the MOSCATO-01 and I-PREDICT trials.^78^, ^79^, ^80^ Although the number of patients with BTC was small in both studies, there is no reason to believe that the results cannot be translated to this subpopulation as well. The ongoing SAFIR ABC-10 trial (NCT05615818), which compares targeted therapy as maintenance treatment after four cycles of standard-of-care chemotherapy versus the continuation of standard treatment in patients with druggable alterations, will provide a definite answer in BTC.

The European Society for Medical Oncology (ESMO) Precision Medicine Working Group recommended tumour next-generation sequencing (NGS) for CCA in 2020, re-endorsed in their 2024 update.^15^^,^^16^ Similarly, the ESMO Biliary Tract Cancer Clinical Practice Guideline strongly recommended molecular profiling for patients with advanced disease.^14^

Parallel sequencing of multiple genes using focused NGS is preferred over single-gene testing. This is the most efficient method for detecting genetic alterations that have low individual prevalence of 1–10%, but altogether total about 30–40% for eCCA and 40–50% for iCCA.^16^

NGS can be performed on formalin-fixed and paraffin-embedded tumour tissue. Alternatively, liquid biopsies using cell-free circulating DNA may be considered, if there is insufficient tumour tissue available for NGS, although the lack of data regarding the DNA shredding of BTC represents a limitation. Nonetheless, liquid biopsy will continue to represent a useful tool to identify molecular alterations in the absence of tissue and monitor response to targeted therapy in a longitudinal fashion. However, the usefulness of circulating tumour (ctDNA) analysis will be limited to a specific set of molecular alterations.

In a large prospective cohort with more than 1000 patients, the utility of ctDNA has been proven with concordance rates between tissue and plasma >85% in BTC-relevant mutations like *IDH1* and *BRAF V600E*, a result that has been confirmed in other cohorts as well.^81^^,^^82^ However, these data are not translatable to *FGFR2* fusions, for which the concordance rate was only 18%.^81^ More recent technologies, like the Illumina panel used in the FOENIX-CCA2 trial, have demonstrated better concordance rates between tissue and plasma (≥80%), but they need to be validated in other cohorts.^11^

Currently, the gene panel should include, at a minimum, the coding DNA regions of *IDH1*, *HER2*, and *BRAF* to test for hotspot mutations. However, the rapidly evolving landscape of drug targets and predictive biomarkers may soon necessitate larger panels and additional analyses as detailed in the next paragraph. For tissue-based testing, gene fusions involving the *FGFR2*, *RET*, and *NTRK* genes should preferably be interrogated at the RNA level using a panel-based method that can identify fusion transcripts of known and unknown fusion partners.^16^ *HER2* amplification should be tested by IHC, looking for a 3+ expression as a biomarker of response to anti-*HER2* therapies.

MSI status can be inferred through an IHC test evaluating tumour tissue expression of MLH1, MSH2, MSH6, and PMS2. Alternatively, DNA-based assays analysing the composition and length of microsatellites can be used.^16^ The combination of the two techniques can reduce the rate of false positives with better identification of good responders to immunotherapy.^83^

The preferred technology depends on the targets and the availability of material for testing [e.g., tissue or ctDNA]. Consultation with a molecular pathologist or the molecular tumour board is strongly recommended.^16^

## Availability and accessibility of molecular profiling and new therapies in Europe

Several factors have hampered the introduction of NGS technologies into routine practice across Europe^84^^,^^85^: (i) access to NGS technologies is greater in countries with a public national reimbursement system^84^^,^^85^; (ii) Central and Eastern European countries lack adequate testing infrastructure, technical implementation, and planned training programmes for laboratory personnel^86^; (iii) more than two-thirds of the countries have no national initiative for genomic testing^85^; (iv) a quality control testing system is often missing and, in many countries, laboratories are using custom-made panels^84^^,^^87^; (v) in some European countries, patients must pay all or part of the cost of biomarker tests, limiting availability of diagnostic and therapeutic options to wealthier individuals.^84^^,^^88^ As a result, NGS testing distribution and application are inconsistent and highly heterogeneous across different countries, with application of NGS being highest in Western and Northern Europe,^84^^,^^87^ whereas in several countries, availability is still limited to clinical trials or basic research. According to our survey, in 25% of the cases, physicians refer to a different institution for genomic profiling. Additionally, in six institutions (17%), molecular sequencing is not even accessible through the national healthcare system, with four of them limiting its use to private practice or research studies (Fig. 1).

Another crucial challenge is the misalignment between the approval of drugs by international agencies and the matched biomarker test price authorisation and reimbursement at the national level. Some countries have tried to address this problem. In Belgium, the so-called Platform CDx includes competencies of the “Commission for Reimbursement of Medicines” and the “Technical Medical Council”; it has been developed to enable an adequate and sustainable biomarker testing practice in the national healthcare system.^89^ In Italy, two separate initiatives of the Ministry of Health on the implementation of NGS technologies for the molecular characterisation of predictive biomarkers for NSCLC and locally advanced/metastatic CCA support the testing on a regional basis.^90^ In Spain, BTC samples can be referred for national NGS assay when local testing is not available, as part of the “Determination of molecular markers in patients included in the Spanish Registry of digestive tumour (RETUD) TTD-20-01” initiative.

Access to new targeted therapies may be similarly impacted at the national/supranational level by the different drug review decisions made by the FDA and the EMA.^91^ EMA is usually characterised by a deferred approval in comparison to FDA (Table 3). As an example, the approval of the three currently available anti-FGFR2 compounds occurred as follows: pemigatinib FDA and EMA approved in 2020 and 2021, respectively; infigratinib FDA approved in 2021, FDA withdrawn in 2022; and futibatinib FDA approved in 2022, EMA approved in 2023.^92^

**Table 3:** Different times of approval of immunotherapy and targeted agents for advanced biliary tract cancers by the EMA and the FDA.

Drug	EMA approval	FDA approval	Difference between FDA and EMA approval (days)
CisGem + durvalumab	December 16th, 2022	September 2nd, 2022	105
CisGem + pembrolizumab	December 11th, 2023	October 31st, 2023	41
Pemigatinib	March 26th, 2021	April 17th, 2020	343
Futibatinib	July 4th, 2023	September 30th, 2022	277
Infigratinib	–	May 28th, 2021 – withdrawn October 5th, 2022	–
Ivosidenib	May 4th, 2023	August 25th, 2021	617
Zanidatamab	July 19th, 2021^a^	–	–
Trastuzumab deruxtecan	–	April 5th, 2024 (agnostic approval)	–
Dabrafenib + trametinib	–	June 22nd, 2022 (agnostic approval)	–
Pembrolizumab	April 25, 2022 (agnostic approval)	May 23rd, 2017 (agnostic approval)	1798
Entrectinib	July 31st, 2020 (agnostic approval)	August 15th, 2019 (agnostic approval)	351
Larotrectinib	September 19th, 2019 (agnostic approval)	November 26th, 2018 (agnostic approval)	297
Selpercatinib	April 29th, 2024 (agnostic approval)	September 21st, 2022 (agnostic approval)	586

Abbreviations: CisGem, cisplatin and gemcitabine; EMA, European Medicines Agency; FDA, Food and Drug Administration.

a Received orphan designation.

As mentioned, there is no direct association between approval by regulatory agencies and reimbursement at the single-nation level.^93^ The only exception in Europe is Germany, where approval implies almost immediate reimbursement.^89^ According to a report of the European Federation of Pharmaceutical Industries and Associations, the average time to reimbursement for innovative treatments is 511 days, ranging from 133 days in Germany to over 899 days in Romania.^94^ This is partly due to the fact that the criteria used for drug reimbursement at the single-nation level can be different from the ones utilised by supranational regulatory bodies. For instance, in the Netherlands, the PASKWIL criteria are applied to determine the health benefit offered by new drugs and are based on a minimal advantage in OS and PFS, something that may impact the marketing authorisation for many BTC drugs.^95^ Additionally, the clinical outcome data need to be included in a wider assessment to specifically define the cost-effectiveness of a new drug in a given population. This will be impacted by the epidemiology of the disease in that given nation, access to matched diagnostic test, cost of management of side effects and complications related to disease progression.

This implies that the availability of medicines varies dramatically across the different European nations and highlights the need for a European community effort to ensure ethical and equal access to novel therapies and related predictive biomarker testing, while also focussing on finding cost-effective solutions to treat patients with advanced BTC.

## Challenges in access to testing and profiling

Access to tumour profiling in BTC presents several challenges, which can be categorised as technical, economic, or systemic barriers.

Technical barriers are related to the intrinsic characteristics of BTC. Given its anatomical location, obtaining sufficient high-quality tissue for molecular analysis can be challenging. Perihilar and distal CCA are often diagnosed by brushing only, with low yield of cellular samples, especially in locally advanced stages in absence of other targetable metastatic lesions.^96^ Pathologists are required to handle tissue specimens carefully to confirm the diagnosis while ensuring tissue conservation for molecular profiling without depleting the sample.^97^ Even when fine needle biopsies are obtained, poor-quality or insufficient DNA/RNA can compromise the accuracy of genomic profiling, due to the low cellularity and the enriched stroma of BTC.^98^ Tumour heterogeneity complicates this issue, as a single biopsy may not be representative of the entire tumour's genomic landscape, which can lead to an incomplete or inaccurate molecular profile. In addition, stroma and tumour microenvironment can play a role in drug response, which is not yet fully understood and may not be captured by NGS. Furthermore, the genomic architecture of BTC can evolve over time and in response to treatments, necessitating repeated profiling, which can be challenging to implement in clinical practice. Liquid biopsy may overcome some of these limitations, but sensitivity may be limited for not-shedding BTC and for detection of fusions, amplifications and large deletions.^99^^,^^100^

From an economic perspective, the costs of comprehensive genomic profiling can be prohibitively high, which currently restricts its use to centres with the necessary financial resources.^82^ Moreover, insurance coverage for genomic profiling is not consistent across different countries or within healthcare systems, often requiring rigorous justification of its clinical utility to secure coverage.^81^ However, it is important to underline the relevance of genomic profiling in guiding therapeutic decisions in BTC and the necessity to efficiently redistribute resources to ensure its widespread application.

Systemic barriers encompass a range of issues, including regulatory hurdles that can delay the approval for new molecular tests and therapies. There is also variability in the standards and practices for genomic profiling, which can result in inconsistencies in test availability and quality.^101^ Moreover, the rapid pace at which the field of cancer genomics is advancing requires regulatory frameworks to stay abreast of new developments, to avoid a lag in the implementation of practices. There is also a need to advocate for equity of access to testing through increased affordability and expansion of insurance coverage, and to educate clinicians about the interpretation and application of genomic data. Addressing these barriers will be critical to fully leverage the potential of precision medicine in improving the outcomes for patients with BTC.

## Resistance to targeted agents

Despite recent advances in targeted therapy for advanced BTC, acquired and intrinsic resistance to targeted therapy remains a significant limitation.

Primary resistance to FGFR inhibitors seems to be associated with co-occurring molecular alterations, such as *TP53* and *CDKN2A*, even though evidence is not yet strong enough to inform clinical practice. Among patients with CCA and *FGFR2* gene fusions treated with selective FGFR inhibitors, secondary *FGFR2* kinase domain mutations are detected in up to 60% of cases, either from post progression ctDNA or tissue biopsy.^102^ These are most commonly found either in the gatekeeper residue, which controls access to the binding pocket, or in the molecular break residue, which forms a hydrogen bond network to reduce kinase activation.^103^ Rarely, acquired mutations affecting the site of covalent binding of the irreversible inhibitor futibatinib have been reported. In the absence of secondary mutations in *FGFR2*, activation of pathways downstream of FGFR2, including PIK3CA, Ras, and Raf, can contribute to primary or secondary resistance, either due to acquired activating mutations, or selection of a pre-existing sub-clonal population. Highly selective FGFR2 inhibitors have demonstrated ability to overcome these on-target resistance mechanisms *in vitro* and *in vivo*, and prospective studies are ongoing in patients with progression on FGFR2 targeted therapy.^41^ An example is tinengotinib, which targets a FGFR2 binding site away from the inner pocket to avoid the impact of sequential mutations.^42^^,^^43^

The mechanisms of primary and secondary resistance to IDH1 inhibitor therapy in *IDH1*-mutant CCA are less well known. In small numbers of patients, secondary gain of function mutations in *IDH2* have been detected following prolonged treatment with ivosidenib, but their biological and clinical significance is not yet clear.^104^ Acquired mutations in *IDH1* have also been described, resulting in impaired binding to ivosidenib and continued production of 2-hydroxyglutarate.^105^ Irreversible inhibitors of mutant *IDH1*, alone or in combination with chemotherapy, have shown promising efficacy in preclinical and early clinical studies in overcoming these on-target resistance mechanisms.^106^^,^^107^

Sequential analysis of ctDNA during treatment with targeted therapy facilitates the longitudinal characterisation of tumour evolution and reflects polyclonal mechanisms of acquired resistance better than analysis of a single biopsy sample.^108^ More widespread access to liquid biopsy could allow the detection of secondary resistance mutations earlier on, allowing the introduction of new drugs that can prolong clinical benefit and the optimisation of the sequencing of the different available targeted drugs. For instance, in patients with *FGFR2* fusions/rearrangements there would be a benefit in identifying the onset of secondary resistance mutations sooner given the presence of new drugs active on clones resistant to first-generation FGFR2 inhibitors.

However, access to high quality ctDNA analysis and to clinical trials of second generation inhibitors varies across countries and socio-economic groups and is a major barrier to improving treatment outcomes.

## Locoregional therapies

Up to 50% of patients with advanced BTC present with locally advanced disease. In this subgroup of patients, local disease control could be improved by combining locoregional treatment (LRT) and systemic therapy,^109^ a goal that is of paramount importance considering that most patients (70%) with iCCA die from intrahepatic disease progression rather than extrahepatic disease.^110^ The benchmark for LRT in iCCA has been set by a subgroup analysis of the ABC trials showing a 3-year OS of 3% for systemic chemotherapy.^111^

The single arm Misphec trial investigated the addition of first line Selective Internal Radiation Therapy (SIRT) to CisGem.^112^ In a propensity-score matched comparison with several clinical trials investigating systemic treatment alone, OS was superior in patients who also received SIRT (HR 0.59; 95% CI, 0.34–0.99, p = 0.049).^113^

Four single-arm phase 2 trials have investigated the benefit of hepatic arterial infusion pump chemotherapy with floxuridine.^114^, ^115^, ^116^, ^117^ The rationale is that floxuridine (a 5-FU analogue) has a 95% first-pass effect in the liver with a 200-fold higher exposure in cancer cells compared to systemic administration. The four trials found an ORR ranging from 38% to 58% with 29%–43% 3-year OS. A prospective randomised trial may provide more definite data to inform clinical practice.

Finally, stereotactic body radiation therapy (SBRT) has been investigated in combination with CisGem in the randomised ABC-07 trial, including 69 patients with locally advanced CCA. No difference in OS was found (HR 1.0; 95% CI, 0.5–1.5, p = 0.63).^118^ However, selected patients may benefit from SBRT in case of lack of progression to induction systemic treatment; more data will be needed to understand if circulating biomarkers (i.e., ctDNA) may provide useful insights in this context.

Access to LRT varies across Europe. Non-randomised trials for LRT showed a promising >5-fold increase in 3-year OS in patients with iCCA.^114^^,^^117^ Randomised controlled trials (e.g., NCT02807181) may help to inform addition of LRT into CCA guidelines and guarantee universal access, but accrual may be challenging due to strict selection criteria and heterogeneity in patient prognosis. Meanwhile, patients with localised BTC should be discussed in a multidisciplinary meeting for consideration of LRT before and after systemic treatment. From our surveys, LRT, such as radiofrequency ablation, transarterial chemoembolisation, and SBRT, are still considered for selected patients only (Fig. 1). It is worth noting that LRT often follow different approval routes, as review of the safety and performance of the devices used is necessary.

## Discussion

The management of BTC has seen a remarkable transformation over the last decade, with the introduction of more than ten novel therapeutic approaches into international recommendations. However, their application in clinical practice is still highly heterogeneous across Europe.

It is interesting to note how some interventions are more rapidly introduced than others. For example, durvalumab received first regulatory approval from the FDA in September 2022, subsequently by the EMA in December 2022, and is already available in 17 countries in Europe after less than two years. Conversely, pemigatinib received FDA approval in April 2020, EMA approval in March 2021 and is not yet widely available over three years later. These discrepancies may be associated with a number of factors that range from the number of patients expected to benefit, the costs associated with the new therapies, the governance pathway of regional authorities, but also the diversified approach to regulatory approvals taken by different industries, and the presence/absence of companion diagnostics associated with the introduction of a novel therapy. It is worth noting that in some countries, the approvals of drugs and companion diagnostics run in parallel (e.g., Germany), while in others, these are independent (e.g., Scotland), making the drug available without having the available diagnostic.

The overall approval pathway includes a first step of interaction with large regulatory bodies, followed by an approval process at country level. In Europe, the first divergence of pathway implementation occurs at this level, where each country follows its own rules. Next, there is an added level of variability within each country, where regional authorities control their own budget and therefore provide their own approval to reimbursability, as also highlighted in our survey.

An overarching collaborative effort of multi-stakeholder consortia can help overcome these disparities. First, inclusion of patients with BTC from different European countries in clinical trials can facilitate recognition of the importance of the new treatment strategy from local authorities. Moreover, real world data reflecting the epidemiology and outcome of BTC in different countries can help local authorities to identify unmet needs. Lastly, a programme of global awareness of the increasing incidence, falling age at diagnosis, and growing impact of BTC on social and economic growth of European countries is essential to promote a more harmonised pathway to approval and implementation of guidelines.

Through the COST Action Precision-BTC-Network CA22125, supported by COST (European Cooperation in Science and Technology), linked to the European Network for the Study of Cholangiocarcinoma (ENSCCA), there is an international effort with representation of several European countries in a coordinated programme of activities aimed to increase education on BTC, support a research programme, and provide a pathway to impact the implementation of innovative strategies in clinical practice. Integrative tasks of these networks are to facilitate discussion with governance agencies to provide an agreed route to approval. The data supporting molecularly targeted therapies in BTC are coming from single arm studies, which decreases the level of confidence for approval at local levels. Indeed, the use of formalised real-world data from multiple European countries for the creation of untreated comparator cohorts with BTC may provide supporting information for the approvals, as long as the path to data collection, cleaning and checking follows standardised rules agreed with the regulatory bodies.

## Conclusion

Treatment options for patients with advanced BTC have recently improved and are expected to further improve in the near future with the approval and reimbursement of novel therapies. However, access to molecular testing and innovative treatments, such as immunotherapy and molecularly targeted agents, is far from homogeneous within Europe, leading to inequalities in healthcare across European countries. Addressing these disparities is critical and requires a global collaborative effort by multiple stakeholder and governance bodies aiming to foster a more harmonised path towards approval, reimbursement, and implementation of innovative strategies in clinical practice. Overcoming challenges in access to testing and treatments and addressing regional disparities will be key to advancing care and improving patient outcomes.

## Contributors

L.R., R.I.R.M., and C.B. contributed to the conceptualisation, supervision, investigation/data acquisition, visualisation, writing–original draft, writing–review & editing. A.F., A.L., A.V., J.E., M.F., M.G.McN., and T.K. contributed to the investigation/data acquisition, writing–original draft, writing–review & editing. A.C.G., A.H., A.U., D.M., E.M., G.M.O’K., J.A., J.B., L.F., M.A.L., T.M., and M.N. contributed to writing–original draft, writing–review & editing.

## Editor note

The Lancet Group takes a neutral position with respect to territorial claims in published maps and institutional affiliations.

## Declaration of interests

L.R. reports grant/research funding (to institution) from AbbVie, Agios, AstraZeneca, BeiGene, Eisai, Exelixis, Fibrogen, Incyte, IPSEN, Jazz Pharmaceuticals, Lilly, MSD, Nerviano Medical Sciences, Roche, Servier, Taiho Oncology, TransThera Sciences, and Zymeworks; consulting fees from AbbVie, AstraZeneca, Basilea, Bayer, Bristol Myers Squibb, Eisai, Elevar Therapeutics, Exelixis, Genenta, Hengrui, Incyte, IPSEN, IQVIA, Jazz Pharmaceuticals, MSD, Nerviano Medical Sciences, Roche, Servier, Taiho Oncology, and Zymeworks; lecture fees from AstraZeneca, Bayer, Bristol Myers Squibb, Guerbet, Incyte, IPSEN, Roche, and Servier; and travel expenses from AstraZeneca. She is chair for the EORTC CITCG HBP/NET Task Force, treasurer for the International Liver Cancer Association, and Special Expert Clinical Trials Europe for NCI GISC Hepatobiliary (HB) Task Force (unpaid positions).

A.L. declares travel and educational support from Ipsen, Pfizer, Bayer, AAA, SirtEx, Novartis, Mylan, Delcath Advanz Pharma, and Roche; speaker honoraria from Merck, Pfizer, Ipsen, Incyte, AAA/Novartis, QED, Servier, AstraZeneca, EISAI, Roche, Advanz Pharma, and MSD; advisory and consultancy honoraria from EISAI, Nutricia, Ipsen, QED, Roche, Servier, Boston Scientific, Albireo Pharma, AstraZeneca, Boehringer Ingelheim, GENFIT, TransThera Biosciences, Taiho, and MSD; principal investigator-associated institutional Funding form QED, Merck, Boehringer Ingelheim, Servier, AstraZeneca, GenFit, Panbela Therapeutics, Novocure GmbH, Camurus AB, Albireo Pharma, Taiho, TransThera, Jazz Therapeutics, and Roche; she is a member of the Knowledge Network and NETConnect Initiatives funded by Ipsen.

G.O.K. declares travel support from MSD, Roche, and Takeda; consulting honoraria from Roche, AstraZeneca, Incyte, Servier; institutional grant support from Roche, AstraZeneca; lecture fees from MSD and Roche.

J.E. receives grants from BMS, Beigene, Boston Scientific, Exeliom biosciences, SUMMIT; consulting fees from MSD, Eisai, BMS, AstraZeneca, Bayer, Roche, Ipsen, Basilea, Merck Serono, Incyte, Servier, Beigene, Taiho, Boston Scientific, Guerbet.

M.M.N. received research grant support to institution from AstraZeneca, Servier, and NuCana; travel and accommodation support from Ipsen, and speaker honoraria from AstraZeneca.

A.V. has consultancy and advisory role for Roche, AstraZeneca, Böhringer-Ingelheim, Ipsen, Incyte, Cogent, EISAI, Zymeworks, Biologix, BMS, Terumo, Elevar, Servier, MSD, Taiho, Jazzpharma, Medivir, Abbvie, Tyra, Falk, Janssen, Lilly; received travel support from Roche, MSD, and Astellas; speaker fees from Roche, AstraZeneca, Böhringer-Ingelheim, Ipsen, Incyte, Cogent, EISAI, Zymeworks, Biologix, BMS, Terumo, Elevar, Servier, MSD, Tahio, Jazzpharma, Medivir, Abbvie, Tyra, Falk, Janssen, Lilly. MF reports grant/research funding (to institution) from Astellas, Roche and Diaceutics; consulting fees from Amgen, Astellas, AstraZeneca, BMS, Diapath, Eli Lilly, Sanofi, GSK, Incyte, IQVIA, Janssen Pharma, MSD, Novartis, Pierre Fabre, Roche, lecture fees from Amgen, Astellas, AstraZeneca, BMS, Diapath, Eli Lilly, GSK, Incyte, IQVIA, Janssen Pharma, Sanofi, MSD, Novartis, Pierre Fabre, Roche. AF declares travel support from AstraZeneca, consulting fees from Taiho, Incyte, and AstraZeneca, and honoraria as speaker from AstraZeneca; he is Scientific Vice-Secretary AEEH.

T.J.K. received consulting fees from Resolution Therapeutics, Clinnovate Health, HistoIndex, Fibrofind, Kynos Therapeutics, Perspectum Diagnostics, Concept Life Sciences; speaker fees from Servier Laboratories, Incyte Corporation, Jazz Pharmaceuticals; he is a committee member of the Pathological Society of Great Britain and Ireland, the British Association for the Study of the Liver, Cholangiocarcinoma-UK, UK Liver Pathology Group.

A.C.G. received consulting fees from AstraZeneca, Bayer, BMS, Eisai, Incyte, Ipsen, IQVIA, MSD, Roche, Servier; lecture fees from AstraZeneca, Bayer, BMS, Eisai, Incyte, Ipsen, Roche, Servier; travel expenses from AstraZeneca and MSD; advisory fees from AstraZeneca, Bayer, BMS, Eisai, Incyte, Ipsen, Roche, Servier; equipment from AstraZeneca.

E.M. reports lecture fees (to the Georgian School of Oncology) from Novartis, Servier, Roche, AstraZeneca; grants from ESMO; travel expenses from ESMO and ESO; she is the director of the Georgian School of Oncology and of the Georgian Society of Geriatric oncology and she is a member of several ESMO committees.

L.F. reports grant/research funding (to institution) from MSD, Bristol Myers Squibb, AstraZeneca, Incyte, BeiGene, Astellas, Daiichi Sankyo, and Roche; consulting fees from MSD, AstraZeneca, Incyte, Taiho Oncology, Servier, Daiichi Sankyo, EliLilly, and BeiGene; lecture fees from Incyte, Bristol Myers Squibb, EliLilly, AstraZeneca, and MSD; travel expenses from Amgen.

A.H. reports consulting fees from Amgen, Sanofi, BMS, Basilea, Incyte, Servier, Relay Therapeutics, Taiho, MSD; lecture fees from Servier, Incyte, Seagen; support for attending meetings from Pierre-Fabre; advisory board fees from Basilea, QED therapeutics, Taiho, Relay Therapeutics, MSD.

M.L. reports research funding to institution from MSD, Exelixis, Amgen, Zymeworks, Basilea, Daiichi Sankyo, Legend Biotech, Genuity Science, consulting role with funding to institution only from AstraZeneca, Astellas pharma, Servier, Roche/Genentech.

T.M. reports consulting or advisory role fees from Ability Pharmaceuticals SL, Arcus Bioscience Inc., AstraZeneca, Basilea Pharma, Baxter, BioLineRX Ltd, Celgene, Eisai, Incyte, Ipsen Bioscience Inc.; grant research funding from MSD, Novocure, QED Therapeutics, Roche Farma, Sanofi-Aventis, Servier, Zymeworks; lecture fees from Janssen, Lilly, Esteve, Daïchi, Biontech, Novartis, Jazz Pharmaceuticals; travel support from Servier, AstraZeneca, Sanofi, Incyte, Lilly, MSD, and Roche.

D.M. reports consulting fees from AbbVie, Amgen, AstraZeneca, Bayer, Bionest Partners, BMS, Incyte, Merck Serono, MSD, Pierre Fabre Oncologie, Roche, Sanofi, Simon-Kutcher & Partners, Servier, Taiho; lecture fees from Amgen, AstraZeneca, Bayer, BMS, Foundation Medicine, Incyte, Leo Pharma, Medscape, Merck Serono, MSD, Pierre Fabre Oncologie, Roche, Sanofi, Servier, Veracyte, Viatris; travel expenses from Amgen, Bayer, BMS, Merck Serono, MSD, Pierre Fabre Oncologie, Roche, Sanofi, Servier, Viatris.

M.N. received grant funding to the GONO foundation from Rising Tide Foundation For Clinical Cancer Research; travel expenses from AstraZeneca; honoraria for lectures or editorial collaboration from Sandoz, Medpoint SRL, Incyte, AstraZeneca, Accademia della Medicina, and Servier; consulting fees from EMD Serono, Basilea Pharmaceutica, Incyte, MSD Italia, Servier, and AstraZeneca; advisory fees from Servier, AstraZeneca, and Taiho.

A.U. received travel support from AstraZeneca.

R.I.R.M. reports institutional funds from AstraZeneca, Incyte, Servier, Taiho and Jazz Pharmaceuticals.

C.B. received honoraria as speaker from AstraZeneca, Incyte, and Servier; honoraria as consultant from Incyte, Servier, Boehringer Ingelheim, AstraZeneca, Jazz Pharmaceutical, Taiho, Molecular Partners, received research funds from Avacta, Medannex, Servier, and her spouse is an employee of AstraZeneca.

All the other authors have no competing interests to declare.
