# Supplementary material for: New systemic treatment paradigms in advanced biliary tract cancer and variations in patient access across Europe
Source: Lancet Reg Health Eur. 2025 Feb 19;50:101170. doi: 10.1016/j.lanepe.2024.101170 (PMC11910789; doi:10.1016/j.lanepe.2024.101170)
Supplement: Supplementary Table S1 [file mmc1.docx]

**Supplementary Material.**

**New systemic treatment paradigms in advanced biliary tract cancer and variations in patient access across Europe: A clinician’s perspective.**

**Table S1. Survey of clinical practice in biliary tract cancer in Europe carried out using google forms.**

| **Question** | **Options for participants** |
| --- | --- |
| Please, indicate your clinical specialty |  |
| Type of centre | Academic  Community |
| Your location - city/town |  |
| Your location - country |  |
| Do you apply the ESMO guidelines in clinical practice? | Yes  No |
| If not, which guidelines do you use? | National guidelines  Other, please specify |
| At your centre, do you have: | HPB surgeon (including potential evaluation for liver transplant)  Dedicated medical oncologists/gastroenterologists  Dedicated tumour board  Other, please specify |
| Do you always proceed with pathological diagnosis before any non-surgical treatment? | Yes  No  Other, please specify |
| Do you have access to molecular profiling? | Yes, at my center  Yes, at another center  No  Other, please specify |
| If you use genomic testing, within clinical practice or research, do you always ask for molecular profiling for all patients with BTC? | At diagnosis, any stage  At advanced stage, for patients who start first line systemic treatment  After first line systemic treatment  Other, please specify |
| Which type of molecular testing do you most frequently use? | NGS – DNA and RNA  NGS DNA  Single gene testing (IHC, FISH, other)  Other, please specify |
| If you ask for NGS, which panel do you use? (how many genes, which platform) |  |
| Do you consider liver transplant for patients with iCCA? | Yes  No  Other, please specify |
| Do you prescribe adjuvant capecitabine after radical surgery? | Yes, to all patients  In selected cases (specify)  No  Other, please specify |
| Do you consider adjuvant radiotherapy after chemotherapy in patients with eCCA? | Yes  No  Other, please specify |
| Do you have access to RFA, TACE, TARE, SBRT for patients with BTC? | Yes  No  Only some of them in selected cases  Other, please specify |
| Which percentage of your patients with advanced disease receive pSACT (First line)? |  |
| Which percentage of your patients with advanced disease receive pSACT (Second line)? |  |
| Which percentage of your patients with advanced disease receive pSACT(Third line)? |  |
| Do you have access to immunotherapy in combination with chemotherapy? | Yes, CisGemDurva  Yes, CiSGemPembro  Yes, both  No  Other, please specify |
| Do you have access to molecularly targeted agents outside research? (multiple choice) | Yes, per ESMO guidelines  Yes, pemigatinib  Yes, futibatinib  Yes, ivosidenib  Other, please specify  No |
| Which is your preferred second line treatment for patients with no actionable alterations? (Or if you don’t have access to targeted agents in your country) | mFOLFOX  FOLFIRI  5-FU + FA + liposomal irinotecan  Other, please specify |
| Do you have access to clinical trials for patients with BTC? | Yes, at your center  Yes, at another center  No  Other, please specify |
| Do you have access to simultaneous care/ palliative care for patients with BTC? | Yes  No  Other, please specify |
| Are patients with BTC referred to a psychologist? | Yes  No  Other, please specify |
| Are patients referred to a dietician? | Yes  No  Other, please specify |
| Are patients put in contact with patient associations? | Yes  No  Other, please specify |
| Please add any comments regarding your clinical practice that you feel are relevant to you and that have not been reflected in the questions above |  |

Abbreviations: 5-FU, 5-fluorouracil; BTC, biliary tract cancer; CisGemDurva, cisplatin + gemcitabine + durvalumab; CisGemPembro, cisplatin + gemcitabine + pembrolizumab; eCCA, extrahepatic cholangiocarcinoma; ESMO, European Society of Medical Oncology; FA, folinic acid; FISH, fluorescence *in situ* hybridisation; FOLFIRI, 5-FU + folinic acid + irinotecan; HPB, HepatoPancreatoBiliary; iCCA, intrahepatic cholangiocarcinoma; IHC, immunohistochemistry; mFOLFOX, 5-FU + folinic acid + oxaliplatin; NGS, next-generation sequencing; pSACT, palliative systemic anticancer treatment; RFA, radiofrequency ablation; SBRT, sterotactic body radiation therapy; TACE, transarterial chemoembolisation; TARE, transarterial radioembolization.
